# Supplementary material for: Best Practices and Progress in Precision-Cut Liver Slice Cultures
Source: Int J Mol Sci. 2021 Jul 1;22(13):7137. doi: 10.3390/ijms22137137 (PMC8267882; doi:10.3390/ijms22137137)
Supplement: Supplementary file 1 [file ijms-22-07137-s001.zip › ijms-1261870-supplementary.pdf]

Supplementary Table S1. Analyzed PCLS publications and parameters used for statistics.

Author	***⌀ (mm)***	***↕ (μm)***	***Slicer***	***Species***	***Time (h)***	***Disease***
Midwoud 2011[1]	4	200-300	Krumdieck	Rat	72	Characterization<br>Metabolization
Lemaire 2011[2]	8	< 200	Krumdieck	Fish	< 24	Toxicology<br>Metabolization
Khan 2011[3]	8	200-300	Krumdieck	Rat<br>Human	24	Toxicology<br>Metabolization
Kasper 2011[4]	8	200-300	Vitron	Porcine	48	Optimization
Elferink 2011[5]	8	200-300	Krumdieck	Human	24	Metabolization
Burkhardt 2011[6]	8	200-300	Vitron	Rat	24	Metabolization
Bie 2011[7]	ns	ns	ns	Mouse	< 24	Steatosis
Thomes 2012[8]	8	200-300	Vitron	Rat	24	Alcoholic liver disease<br>Metabolization
Rijk 2012[9]	8	200-300	Krumdieck	Bovine	< 24	Metabolization
Lemaire 2012[10]	8	< 200	Krumdieck	Fish	< 24	Environmental toxicity
Guo 2012[11]	8	200-300	Vibratome	Rat	< 24	Fibrosis
Guan 2012[12]	5	200-300	Krumdieck	Rat	< 24	Optimization
Dragoni 2012[13]	8	200-300	Krumdieck	Rat	24	Toxicity
Wang 2013[14]	8	200-300	ns	Rat	24	Fibrosis
Szalowska 2013[15]	5	200-300	Krumdieck	Mouse	24	Cholestasis

						Toxicity
Szalowska 2013[16]	5	200-300	Krumdieck	Mouse	24	Optimization
Moran-Salvador 2013[17]	Other	200-300	Vibratome	Mouse	< 24	Fibrosis<br>Inflammation
Morales-Ibanez 2013[18]	ns	200-300	Vibratome	Mouse	48	Alcoholic liver disease
Karim 2013[19]	ns	200-300	Krumdieck	Human	48	Alcoholic liver disease
Hadi 2013[20]	5	200-300	Krumdieck	Human	24	Toxicity
Hadi 2013[21]	5	200-300	Krumdieck	Mouse<br>Rat<br>Human	24	Toxicity
Guan 2013[22]	5	200-300	Krumdieck	Rat	< 24	Optimization
Duryee 2014[23]	8	200-300	Vitron	Rat	72	Alcoholic liver disease<br>Fatty liver disease
Westra 2014[24]	5	200-300	Krumdieck	Rat	48	Fibrosis
Westra 2014[25]	5	200-300	Krumdieck	Rat	72	Fibrosis
Van Swelm 2014[26]	5	200-300	Krumdieck	Mouse<br>Rat	48	Toxicity
Szalowska 2014[27]	5	200-300	Krumdieck	Mouse	24	Steatosis
Szalowska 2014[28]	5	200-300	Krumdieck	Mouse	24	Compound testing
Rius 2014[29]	Other	200-300	Vibratome	Mouse	24	Steatosis
Kwon 2014[30]	8	200-300	Vitron	Mouse	72	Alcoholic liver disease<br>Steatosis<br>Inflammation<br>Fibrosis

Koch 2014[31]	Other	200-300	Krumdieck	Mouse	72	Cancer
				Rat		
Jetten 2014[32]	4	< 200	Krumdieck	Human	24	Metabolization
Fredriksson 2014[33]	4	200-300	Krumdieck	Human	24	Toxicity
Eide 2014[34]	8	200-300	Other	Fish	48	Toxicity
Vatakuti 2015[35]	8	200-300	Krumdieck	Rat	24	Toxicity<br>Fibrosis
Starokozhko 2015[36]	5	200-300	Krumdieck	Rat	> 72	Optimization
Sadasivan 2015[37]	Other	< 200	Vibratome	Mouse	48	Fibrosis
Page 2015[38]	ns	ns	ns	Rat	72	Alcoholic liver disease
Okun 2015[39]	8	200-300	Krumdieck	Mouse	< 24	Other
Mattei 2015[40]	Other	200-300	Vibratome	Porcine	< 24	Characterization
Maté 2015[41]	8	200-300	Brendel	Rat	< 24	Metabolization
	10			Bovine		
Liu 2015[42]	Other	200-300	Other	Fish	< 24	Toxicity
Janssen 2015[43]	5	200-300	Krumdieck	Human	24	Compound testing
Du 2015[44]	Other	200-300	Other	Fish	< 24	Toxicity
Daum 2015[45]	5	200-300	Krumdieck	Rat	48	Toxicity
Westra 2016[46]	5	200-300	Krumdieck	Human	48	Fibrosis
Viviani 2017[47]	10	200-300	Brendel/Vitron	Bovine	< 24	Toxicity
Vatakuti 2017[48]	5	200-300	Krumdieck	Human	24	Cholestasis

Vatakuti 2016[49]	5	200-300	Krumdieck	Human	24	Toxicity
Starokozhko 2017[50]	5	200-300	Krumdieck	Human	> 72	Optimization
Iswandana 2016[51]	5	> 300	Krumdieck	Rat<br>Mouse	48	Toxicity<br>Fibrosis<br>Inflammation
Ijssennagger 2016[52]	5	200-300	Krumdieck	Human<br>Mouse	24	Compound testing
Coombes 2016[53]	ns	200-300	Krumdieck	Mouse	48	NASH<br>Fibrosis
Bizarro 2016[54]	8	200-300	Other	Fish	48	Toxicity
Vilaseca 2017[55]	ns	200-300	Vibratome	Human	24	Cirrhosis
Pushparajah 2017[56]	8	200-300	Krumdieck	Rat	24	Metabolization
Villalobos-Garcia 2017[57]	8	> 300	Krumdieck	Rat	< 24	Alcoholic liver disease
Starokozhko 2017[58]	5	200-300	Krumdieck	Rat	48	Cholestasis
de Mesquita 2017[59]	ns	200-300	Vibratome	Human	24	Cirrhosis
Fortin 2017[60]	5	200-300	Vibratome	Bovine	72	Steatosis
Luangmonkong 2017[61]	8	200-300	Krumdieck	Human<br>Rat	48<br>72	Fibrosis
Granitzny 2017[62]	8	200-300	Krumdieck	Rat	24	Toxicity
Cao 2017[63]	Other	200-300	Other	Fish	< 24	Toxicity<br>Inflammation
Viviani 2018[64]	10	200-300	Brendel/Vitron	Bovine	< 24	Metabolization
Yadetie 2018[65]	8	200-300	Vibratome	Fish	48	Toxicity

Stuchlikova 2018[66]	8	< 200	Krumdieck	Human	24	Metabolization
Rodriguez 2018[67]	ns	ns	ns	Human	ns	Compound testing
Starokozhko 2018[68]	8	200-300	Krumdieck	Human	24	Other
Quesnot 2018[69]	ns	ns	Vibratome	Human	ns	Metabolization
Wu 2018[70]	6	200-300	Vibratome	Human	> 72	Characterization<br>Immunology
Rius 2018[71]	Other	200-300	Vibratome	Mouse	24	Steatosis
Prins 2019[72]	8	200-300	Krumdieck	Rat	48	NAFLD
Lev-Cohain 2019[73]	Other	> 300	Other	Mouse	< 24	Metabolization
Luangmonkong 2018[74]	8	200-300	Krumdieck	Human<br>Rat	48	Fibrosis
Duryee 2018[75]	8	200-300	Brendel/Vitron	Mouse	72	Metabolization<br>Alcoholic liver disease
Beaumont 2018[76]	5	200-300	Krumdieck	Mouse	< 24	Inflammation<br>Steatosis
Maté 2019[77]	10	200-300	Brendel/Vitron	Bovine	< 24	Metabolization
Paish 2019[78]	8	200-300	Vibratome	Human<br>Rat	> 72	Optimization<br>Fibrosis
Stärkel 2019[79]	8	200-300	Krumdieck	Human	72	Alcoholic liver disease<br>Fibrosis
Stefanovic 2019[80]	8	> 300	Krumdieck	Rat	72	Fibrosis
Harvey 2019[81]	Other	200-300	Other	Fish	> 72	Steatosis
De Chiara 2020[82]	8	200-300	Vibratome	Rat	> 72	NAFLD

						Fibrosis
Bigaeva 2019[83]	8	200-300	Krumdieck	Mouse<br>Human	48	Characterization
Gore 2019[84]	5	200-300	Krumdieck	Mouse<br>Human	48	Fibrosis
Gore 2020[85]	5	200-300	Krumdieck	Mouse	48	Fibrosis<br>Inflammation
Bigaeva 2020[86]	6	200-300	Krumdieck	Mouse	48	Fibrosis
Bartucci 2020[87]	5	200-300	Krumdieck	Rat<br>Human	72	Other
Daga 2020[88]	5	200-300	Krumdieck	Rat	24	Toxicity
Palma 2020[89]	5	200-300	Krumdieck	Human	24	Alcoholic liver disease
Zhang 2020[90]	2	200-300	Other	Human	> 72	Optimization
Aoudjehane 2020[91]	5	200-300	Vibratome	Human	24	Steatosis<br>Optimization
Kenerson 2020[92]	4<br>6	200-300	Vibratome	Human	> 72	Cancer<br>Characterization
Van Dijk 2020[93]	8	200-300	Krumdieck	Human	48	Fibrosis
Adhyatmika 2020[94]	5	200-300	Krumdieck	Human<br>Mouse	48	Fibrosis
Suriguga 2020[95]	6	200-300	Krumdieck	Mouse	48	Inflammation/Immunology
Serrano 2021[96]	8	200-300	Krumdieck	Fish	48	Metabolization
Shepherd 2020[97]	8	200-300	Krumdieck	Mouse	48	Steatosis
Barcena-Varela 2021[98]	8	200-300	Vibratome	Human	24	Fibrosis

Gupta 2021[99]	8	200-300	Krumdieck	Human	24	Other
Duran-Guëll 2021[100]	5	200-300	Vibratome	Mouse	Other	Toxicity
Bartucci 2021[101]	5	200-300<br>> 300	Krumdieck	Mouse	72	Inflammation<br>Fibrosis
Aluru 2021[102]	Other	ns	ns	Fish	48	Metabolization
Aranguren-Abadia 2020[103]	8	200-300	Vibratome	Fish	48	Characterization
Estrada-Ortiz 2019[104]	5	200-300	Krumdieck	Rat	24	Cancer
Guzman-Delgado 2019[105]	5	200-300	Krumdieck	Hamster	24	Other
Tian 2020[106]	5	200-300	Vibratome	Rat	24	Cancer
Zarybnicky 2020[107]	8	< 200	Krumdieck	Human	24	Optimization
Total	*107*					

ns = not specified

1. van Midwoud, P.M., et al., *Hydrogel embedding of precision-cut liver slices in a microfluidic device improves drug metabolic activity.* Biotechnol Bioeng, 2011. **108**(6): p. 1404-12.
2. Lemaire, B., et al., *Precision-Cut Liver Slices of Salmo salar as a tool to investigate the oxidative impact of CYP1A-mediated PCB 126 and 3-methylcholanthrene metabolism.* Toxicol In Vitro, 2011. **25**(1): p. 335-42.
3. Khan, A.A., et al., *The role of lithocholic acid in the regulation of bile acid detoxication, synthesis, and transport proteins in rat and human intestine and liver slices.* Toxicol In Vitro, 2011. **25**(1): p. 80-90.
4. Kasper, H.U., et al., *Cryopreservation of precision cut tissue slices (PCTS): investigation of morphology and reactivity.* Exp Toxicol Pathol, 2011. **63**(6): p. 575-80.
5. Elferink, M.G., et al., *Gene expression analysis of precision-cut human liver slices indicates stable expression of ADME-Tox related genes.* Toxicol Appl Pharmacol, 2011. **253**(1): p. 57-69.

6. Burkhardt, B., et al., *Oxidative metabolism of the mycotoxins alternariol and alternariol-9-methyl ether in precision-cut rat liver slices in vitro.* Mol Nutr Food Res, 2011. **55**(7): p. 1079-86.
7. Bie, J., et al., *Macrophage-specific transgenic expression of cholesteryl ester hydrolase attenuates hepatic lipid accumulation and also improves glucose tolerance in ob/ob mice.* Am J Physiol Endocrinol Metab, 2012. **302**(10): p. E1283-91.
8. Thomes, P.G., et al., *Proteasome activity and autophagosome content in liver are reciprocally regulated by ethanol treatment.* Biochem Biophys Res Commun, 2012. **417**(1): p. 262-7.
9. Rijk, J.C., et al., *Bovine liver slices: A multifunctional in vitro model to study the prohormone dehydroepiandrosterone (DHEA).* Toxicol In Vitro, 2012. **26**(6): p. 1014-21.
10. Lemaire, B., et al., *Precision-cut liver slices to investigate responsiveness of deep-sea fish to contaminants at high pressure.* Environ Sci Technol, 2012. **46**(18): p. 10310-6.
11. Guo, Y., et al., *Protective effect of sodium ferulate on acetaldehyde-treated precision-cut rat liver slices.* J Med Food, 2012. **15**(6): p. 557-62.
12. Guan, N., et al., *Effects of cryoprotectant addition and washout methods on the viability of precision-cut liver slices.* Cryobiology, 2012. **65**(3): p. 179-87.
13. Dragoni, S., et al., *Gold nanoparticles uptake and cytotoxicity assessed on rat liver precision-cut slices.* Toxicol Sci, 2012. **128**(1): p. 186-97.
14. Wang, K., et al., *Hepatic apoptosis can modulate liver fibrosis through TIMP1 pathway.* Apoptosis, 2013. **18**(5): p. 566-77.
15. Szalowska, E., et al., *Treatment of mouse liver slices with cholestatic hepatotoxicants results in down-regulation of Fxr and its target genes.* BMC Med Genomics, 2013. **6**: p. 39.
16. Szalowska, E., et al., *Effect of oxygen concentration and selected protocol factors on viability and gene expression of mouse liver slices.* Toxicol In Vitro, 2013. **27**(5): p. 1513-24.
17. Morán-Salvador, E., et al., *Cell-specific PPARγ deficiency establishes anti-inflammatory and anti-fibrogenic properties for this nuclear receptor in non-parenchymal liver cells.* J Hepatol, 2013. **59**(5): p. 1045-53.
18. Morales-Ibanez, O., et al., *Human and experimental evidence supporting a role for osteopontin in alcoholic hepatitis.* Hepatology, 2013. **58**(5): p. 1742-56.
19. Karim, S., et al., *An in vitro model of human acute ethanol exposure that incorporates CXCR3- and CXCR4-dependent recruitment of immune cells.* Toxicol Sci, 2013. **132**(1): p. 131-41.
20. Hadi, M., et al., *Human precision-cut liver slices as an ex vivo model to study idiosyncratic drug-induced liver injury.* Chem Res Toxicol, 2013. **26**(5): p. 710-20.
21. Hadi, M., et al., *AMAP, the alleged non-toxic isomer of acetaminophen, is toxic in rat and human liver.* Arch Toxicol, 2013. **87**(1): p. 155-65.
22. Guan, N., et al., *Analysis of gene expression changes to elucidate the mechanism of chilling injury in precision-cut liver slices.* Toxicol In Vitro, 2013. **27**(2): p. 890-9.

23. Duryee, M.J., et al., *Precision-cut liver slices from diet-induced obese rats exposed to ethanol are susceptible to oxidative stress and increased fatty acid synthesis.* Am J Physiol Gastrointest Liver Physiol, 2014. **306**(3): p. G208-17.
24. Westra, I.M., et al., *The effect of antifibrotic drugs in rat precision-cut fibrotic liver slices.* PLoS One, 2014. **9**(4): p. e95462.
25. Westra, I.M., et al., *Precision-cut liver slices as a model for the early onset of liver fibrosis to test antifibrotic drugs.* Toxicol Appl Pharmacol, 2014. **274**(2): p. 328-38.
26. van Swelm, R.P., et al., *Proteomic profiling in incubation medium of mouse, rat and human precision-cut liver slices for biomarker detection regarding acute drug-induced liver injury.* J Appl Toxicol, 2014. **34**(9): p. 993-1001.
27. Szalowska, E., et al., *Model steatogenic compounds (amiodarone, valproic acid, and tetracycline) alter lipid metabolism by different mechanisms in mouse liver slices.* PLoS One, 2014. **9**(1): p. e86795.
28. Szalowska, E., et al., *Transcriptomic signatures of peroxisome proliferator-activated receptor α (PPARα) in different mouse liver models identify novel aspects of its biology.* BMC Genomics, 2014. **15**(1): p. 1106.
29. Rius, B., et al., *Resolvin D1 primes the resolution process initiated by calorie restriction in obesity-induced steatohepatitis.* Faseb j, 2014. **28**(2): p. 836-48.
30. Kwon, H.J., et al., *Aldehyde dehydrogenase 2 deficiency ameliorates alcoholic fatty liver but worsens liver inflammation and fibrosis in mice.* Hepatology, 2014. **60**(1): p. 146-57.
31. Koch, A., et al., *Murine precision-cut liver slices (PCLS): a new tool for studying tumor microenvironments and cell signaling ex vivo.* Cell Commun Signal, 2014. **12**: p. 73.
32. Jetten, M.J., et al., *Interindividual variation in response to xenobiotic exposure established in precision-cut human liver slices.* Toxicology, 2014. **323**: p. 61-9.
33. Fredriksson, L., et al., *Drug-induced endoplasmic reticulum and oxidative stress responses independently sensitize toward TNFα-mediated hepatotoxicity.* Toxicol Sci, 2014. **140**(1): p. 144-59.
34. Eide, M., et al., *Precision-cut liver slices of Atlantic cod (Gadus morhua): an in vitro system for studying the effects of environmental contaminants.* Aquat Toxicol, 2014. **153**: p. 110-5.
35. Vatakuti, S., et al., *Acute toxicity of CCl4 but not of paracetamol induces a transcriptomic signature of fibrosis in precision-cut liver slices.* Toxicol In Vitro, 2015. **29**(5): p. 1012-20.
36. Starokozhko, V., et al., *Viability, function and morphological integrity of precision-cut liver slices during prolonged incubation: Effects of culture medium.* Toxicol In Vitro, 2015. **30**(1 Pt B): p. 288-99.
37. Sadasivan, S.K., et al., *Developing an in vitro screening assay platform for evaluation of antifibrotic drugs using precision-cut liver slices.* Fibrogenesis Tissue Repair, 2015. **8**(1): p. 1.
38. Page, A., et al., *Alcohol directly stimulates epigenetic modifications in hepatic stellate cells.* J Hepatol, 2015. **62**(2): p. 388-97.

39. Okun, J.G., et al., *Molecular regulation of urea cycle function by the liver glucocorticoid receptor*. Mol Metab, 2015. **4**(10): p. 732-40.
40. Mattei, G., et al., *Profile analysis of hepatic porcine and murine brain tissue slices obtained with a vibratome.* PeerJ, 2015. **3**: p. e932.
41. Maté, M.L., et al., *Gene expression and enzyme function of two cytochrome P450 3A isoenzymes in rat and cattle precision cut liver slices.* Xenobiotica, 2015. **45**(7): p. 563-70.
42. Liu, Y., et al., *Protective effects of Lycium barbarum polysaccharides against carbon tetrachloride-induced hepatotoxicity in precision-cut liver slices in vitro and in vivo in common carp (Cyprinus carpio L.).* Comp Biochem Physiol C Toxicol Pharmacol, 2015. **169**: p. 65-72.
43. Janssen, A.W., et al., *The impact of PPARα activation on whole genome gene expression in human precision cut liver slices.* BMC Genomics, 2015. **16**: p. 760.
44. Du, J.L., et al., *A Study of 2,3,7,8-Tetrachlorodibenzo-p-dioxin Induced Liver Injury in Jian Carp (Cyprinus carpio var. Jian) Using Precision-Cut Liver Slices.* Bull Environ Contam Toxicol, 2016. **96**(1): p. 55-61.
45. Daum, S., et al., *Improved synthesis of N-benzylaminoferrocene-based prodrugs and evaluation of their toxicity and antileukemic activity.* J Med Chem, 2015. **58**(4): p. 2015-24.
46. Westra, I.M., et al., *Human precision-cut liver slices as a model to test antifibrotic drugs in the early onset of liver fibrosis.* Toxicol In Vitro, 2016. **35**: p. 77-85.
47. Viviani, P., et al., *Assessment of liver slices for research on metabolic drug-drug interactions in cattle.* Xenobiotica, 2017. **47**(11): p. 933-942.
48. Vatakuti, S., et al., *Validation of precision-cut liver slices to study drug-induced cholestasis: a transcriptomics approach.* Arch Toxicol, 2017. **91**(3): p. 1401-1412.
49. Vatakuti, S., et al., *Classification of Cholestatic and Necrotic Hepatotoxicants Using Transcriptomics on Human Precision-Cut Liver Slices.* Chem Res Toxicol, 2016. **29**(3): p. 342-51.
50. Starokozhko, V., et al., *Maintenance of drug metabolism and transport functions in human precision-cut liver slices during prolonged incubation for 5 days.* Arch Toxicol, 2017. **91**(5): p. 2079-2092.
51. Iswandana, R., et al., *Organ- and species-specific biological activity of rosmarinic acid.* Toxicol In Vitro, 2016. **32**: p. 261-8.
52. Ijssennagger, N., et al., *Gene expression profiling in human precision cut liver slices in response to the FXR agonist obeticholic acid.* J Hepatol, 2016. **64**(5): p. 1158-1166.
53. Coombes, J.D., et al., *Osteopontin is a proximal effector of leptin-mediated non-alcoholic steatohepatitis (NASH) fibrosis.* Biochim Biophys Acta, 2016. **1862**(1): p. 135-44.
54. Bizarro, C., et al., *Single and mixture effects of aquatic micropollutants studied in precision-cut liver slices of Atlantic cod (Gadus morhua).* Aquat Toxicol, 2016. **177**: p. 395-404.
55. Vilaseca, M., et al., *Mitochondria-targeted antioxidant mitoquinone deactivates human and rat hepatic stellate cells and reduces portal hypertension in cirrhotic rats.* Liver Int, 2017. **37**(7): p. 1002-1012.

56. Pushparajah, D., D. Lewis, and C. Ioannides, *Up-regulation of CYP1A1 and phase II enzymes by 5-ring isomeric polycyclic aromatic hydrocarbons in precision-cut rat hepatic slices: Importance of molecular shape.* Toxicol In Vitro, 2017. **40**: p. 203-213.
57. Villalobos-García, D. and R. Hernández-Muñoz, *Catalase increases ethanol oxidation through the purine catabolism in rat liver.* Biochem Pharmacol, 2017. **137**: p. 107-112.
58. Starokozhko, V., et al., *Rat precision-cut liver slices predict drug-induced cholestatic injury.* Arch Toxicol, 2017. **91**(10): p. 3403-3413.
59. de Mesquita, F.C., et al., *Liraglutide improves liver microvascular dysfunction in cirrhosis: Evidence from translational studies.* Sci Rep, 2017. **7**(1): p. 3255.
60. Fortin, É., et al., *Linoleic acid, α-linolenic acid and enterolactone affect lipid oxidation and expression of lipid metabolism and antioxidant-related genes in hepatic tissue of dairy cows.* Br J Nutr, 2017. **117**(9): p. 1199-1211.
61. Luangmonkong, T., et al., *Evaluating the antifibrotic potency of galunisertib in a human ex vivo model of liver fibrosis.* Br J Pharmacol, 2017. **174**(18): p. 3107-3117.
62. Granitzny, A., et al., *Maintenance of high quality rat precision cut liver slices during culture to study hepatotoxic responses: Acetaminophen as a model compound.* Toxicol In Vitro, 2017. **42**: p. 200-213.
63. Cao, L., et al., *Anti-inflammatory and hepatoprotective effects of glycyrrhetinic acid on CCl(4)-induced damage in precision-cut liver slices from Jian carp (Cyprinus carpio var. jian) through inhibition of the nf-kB pathway.* Fish Shellfish Immunol, 2017. **64**: p. 234-242.
64. Viviani, P., et al., *Assessment of the pharmacological interactions between the nematodicidal fenbendazole and the flukicidal triclabendazole: In vitro studies with bovine liver microsomes and slices.* J Vet Pharmacol Ther, 2018. **41**(3): p. 476-484.
65. Yadetie, F., et al., *RNA-Seq analysis of transcriptome responses in Atlantic cod (Gadus morhua) precision-cut liver slices exposed to benzo[a]pyrene and 17α-ethynylestradiol.* Aquat Toxicol, 2018. **201**: p. 174-186.
66. Raisová Stuchlíková, L., et al., *The metabolism of flubendazole in human liver and cancer cell lines.* Drug Test Anal, 2018.
67. de la Rosa Rodriguez, M.A., et al., *The whole transcriptome effects of the PPARα agonist fenofibrate on livers of hepatocyte humanized mice.* BMC Genomics, 2018. **19**(1): p. 443.
68. Starokozhko, V., et al., *Differentiation of human-induced pluripotent stem cell under flow conditions to mature hepatocytes for liver tissue engineering.* J Tissue Eng Regen Med, 2018. **12**(5): p. 1273-1284.
69. Quesnot, N., et al., *Production of chlorzoxazone glucuronides via cytochrome P4502E1 dependent and independent pathways in human hepatocytes.* Arch Toxicol, 2018. **92**(10): p. 3077-3091.
70. Wu, X., et al., *Precision-cut human liver slice cultures as an immunological platform.* J Immunol Methods, 2018. **455**: p. 71-79.
71. Rius, B., et al., *The specialized proresolving lipid mediator maresin 1 protects hepatocytes from lipotoxic and hypoxia-induced endoplasmic reticulum stress.* Faseb j, 2017. **31**(12): p. 5384-5398.
72. Prins, G.H., et al., *A Pathophysiological Model of Non-Alcoholic Fatty Liver Disease Using Precision-Cut Liver Slices.* Nutrients, 2019. **11**(3).

73. Lev-Cohain, N., et al., *Real-time ALT and LDH activities determined in viable precision-cut mouse liver slices using hyperpolarized [1-(13) C]pyruvate-Implications for studies on biopsied liver tissues.* NMR Biomed, 2019. **32**(2): p. e4043.
74. Luangmonkong, T., et al., *In vitro and ex vivo anti-fibrotic effects of LY2109761, a small molecule inhibitor against TGF-β.* Toxicol Appl Pharmacol, 2018. **355**: p. 127-137.
75. Duryee, M.J., et al., *Liver tissue metabolically transformed by alcohol induces immune recognition of liver self-proteins but not in vivo inflammation.* Am J Physiol Gastrointest Liver Physiol, 2018. **314**(3): p. G418-g430.
76. Beaumont, M., et al., *The gut microbiota metabolite indole alleviates liver inflammation in mice.* Faseb j, 2018. **32**(12): p. fj201800544.
77. Maté, L., et al., *Effects of fenbendazole and triclabendazole on the expression of cytochrome P450 1A and flavin-monooxygenase isozymes in bovine precision-cut liver slices.* Vet J, 2019. **245**: p. 61-69.
78. Paish, H.L., et al., *A Bioreactor Technology for Modeling Fibrosis in Human and Rodent Precision-Cut Liver Slices.* Hepatology, 2019. **70**(4): p. 1377-1391.
79. Stärkel, P., et al., *Deficient IL-6/Stat3 Signaling, High TLR7, and Type I Interferons in Early Human Alcoholic Liver Disease: A Triad for Liver Damage and Fibrosis.* Hepatol Commun, 2019. **3**(7): p. 867-882.
80. Stefanovic, B., et al., *Discovery and evaluation of inhibitor of LARP6 as specific antifibrotic compound.* Sci Rep, 2019. **9**(1): p. 326.
81. Harvey, T.N., et al., *Liver slice culture as a model for lipid metabolism in fish.* PeerJ, 2019. **7**: p. e7732.
82. De Chiara, F., et al., *Ammonia Scavenging Prevents Progression of Fibrosis in Experimental Nonalcoholic Fatty Liver Disease.* Hepatology, 2020. **71**(3): p. 874-892.
83. Bigaeva, E., et al., *Transcriptomic characterization of culture-associated changes in murine and human precision-cut tissue slices.* Arch Toxicol, 2019. **93**(12): p. 3549-3583.
84. Gore, E., et al., *PI3K inhibition reduces murine and human liver fibrogenesis in precision-cut liver slices.* Biochem Pharmacol, 2019. **169**: p. 113633.
85. Gore, E., et al., *Investigating fibrosis and inflammation in an ex vivo NASH murine model.* Am J Physiol Gastrointest Liver Physiol, 2020. **318**(2): p. G336-g351.
86. Bigaeva, E., et al., *Exploring organ-specific features of fibrogenesis using murine precision-cut tissue slices.* Biochim Biophys Acta Mol Basis Dis, 2020. **1866**(1): p. 165582.
87. Bartucci, R., et al., *Time-Resolved Quantification of Nanoparticle Uptake, Distribution, and Impact in Precision-Cut Liver Slices.* Small, 2020. **16**(21): p. e1906523.
88. Daga, M., et al., *Glutathione-responsive cyclodextrin-nanosponges as drug delivery systems for doxorubicin: Evaluation of toxicity and transport mechanisms in the liver.* Toxicol In Vitro, 2020. **65**: p. 104800.

89. Palma, E., et al., *Perturbations in Mitochondrial Dynamics Are Closely Involved in the Progression of Alcoholic Liver Disease.* Alcohol Clin Exp Res, 2020. **44**(4): p. 856-865.
90. Zhang, Y., et al., *Cryopreserved biopsy tissues of rectal cancer liver metastasis for assessment of anticancer drug response in vitro and in vivo.* Oncol Rep, 2020. **43**(2): p. 405-414.
91. Aoudjehane, L., et al., *Novel defatting strategies reduce lipid accumulation in primary human culture models of liver steatosis.* Dis Model Mech, 2020. **13**(4).
92. Kenerson, H.L., et al., *Tumor slice culture as a biologic surrogate of human cancer.* Ann Transl Med, 2020. **8**(4): p. 114.
93. van Dijk, F., et al., *Design of a Gene Panel to Expose the Versatile Role of Hepatic Stellate Cells in Human Liver Fibrosis.* Pharmaceutics, 2020. **12**(3).
94. Adhyatmika, A., et al., *Osteoprotegerin is More than a Possible Serum Marker in Liver Fibrosis: A Study into its Function in Human and Murine Liver.* Pharmaceutics, 2020. **12**(5).
95. Suriguga, S., et al., *Host microbiota dictates the proinflammatory impact of LPS in the murine liver.* Toxicol In Vitro, 2020. **67**: p. 104920.
96. Serrano, J., et al., *In vitro metabolism assessment of thiacloprid in rainbow trout and rat by LC-UV and high resolution-mass spectrometry.* Xenobiotica, 2021: p. 1-13.
97. Shepherd, E.L., et al., *Inhibition of vascular adhesion protein-1 modifies hepatic steatosis in vitro and in vivo.* World J Hepatol, 2020. **12**(11): p. 931-948.
98. Barcena-Varela, M., et al., *Epigenetic mechanisms and metabolic reprogramming in fibrogenesis: dual targeting of G9a and DNMT1 for the inhibition of liver fibrosis.* Gut, 2021. **70**(2): p. 388-400.
99. Gupta, R., et al., *Comparing in vitro human liver models to in vivo human liver using RNA-Seq.* Arch Toxicol, 2021. **95**(2): p. 573-589.
100. Duran-Güell, M., et al., *Albumin protects the liver from tumor necrosis factor α-induced immunopathology.* Faseb j, 2021. **35**(2): p. e21365.
101. Bartucci, R., et al., *Nanoparticle-induced inflammation and fibrosis in ex vivo murine precision-cut liver slices and effects of nanoparticle exposure conditions.* Arch Toxicol, 2021.
102. Aluru, N., et al., *Hepatic gene expression profiling of OPFR exposed Atlantic cod (Gadus morhua) liver revealed altered cholesterol biosynthesis and lipid metabolism.* Environ Toxicol Chem, 2021.
103. Aranguren-Abadía, L., et al., *Molecular and Functional Properties of the Atlantic Cod (Gadus morhua) Aryl Hydrocarbon Receptors Ahr1a and Ahr2a.* Environ Sci Technol, 2020. **54**(2): p. 1033-1044.
104. Estrada-Ortiz, N., et al., *Ex vivo toxicological evaluation of experimental anticancer gold(i) complexes with lansoprazole-type ligands.* Toxicol Res (Camb), 2019. **8**(6): p. 885-895.
105. Guzmán-Delgado, N.E., et al., *Development of a Novel Ex-vivo 3D Model to Screen Amoebicidal Activity on Infected Tissue.* Sci Rep, 2019. **9**(1): p. 8396.

106. Tian, H., et al., *A Novel Tissue-Based Liver-Kidney-on-a-Chip Can Mimic Liver Tropism of Extracellular Vesicles Derived from Breast Cancer Cells.* Biotechnol J, 2020. **15**(2): p. e1900107.
107. Zárybnický, T., et al., *The Selection and Validation of Reference Genes for mRNA and microRNA Expression Studies in Human Liver Slices Using RT-qPCR.* Genes (Basel), 2019. **10**(10).
